# Involvement of Host Stroma Cells and Tissue Fibrosis in Pancreatic Tumor Development in Transgenic Mice

**DOI:** 10.1371/journal.pone.0041833

**Published:** 2012-07-25

**Authors:** Itai Spector, Yael Zilberstein, Adi Lavy, Arnon Nagler, Olga Genin, Mark Pines

**Affiliations:** 1 Institute of Animal Sciences, The Volcani Center, Bet Dagan, Israel; 2 Department of Animal Sciences, Hebrew University of Jerusalem, Rehovot, Israel; 3 The Sackler Cellular and Molecular Imaging Center (SCMIC), Tel Aviv University, Tel Aviv, Israel; 4 Department of Hematology and Bone Marrow Transplantation, Chaim Sheba Medical Center, Tel Hashomer, Israel; University of Bergen, Norway

## Abstract

**Introduction:**

Stroma cells and extracellular matrix (ECM) components provide the pivotal microenvironment for tumor development. The study aimed to evaluate the importance of the pancreatic stroma for tumor development.

**Methods:**

Pancreatic tumor cells were implanted subcutaneously into green fluorescent protein transgenic mice, and stroma cells invading the tumors were identified through immunohistochemistry. Inhibition of tumor invasion by stroma cells was achieved with halofuginone, an inhibitor of TGFβ/Smad3 signaling, alone or in combination with chemotherapy. The origin of tumor ECM was evaluated with species-specific collagen I antibodies and *in situ* hybridization of collagen α1(I) gene. Pancreatic fibrosis was induced by cerulean injection and tumors by spleen injection of pancreatic tumor cells.

**Results:**

Inhibition of stroma cell infiltration and reduction of tumor ECM levels by halofuginone inhibited development of tumors derived from mouse and human pancreatic cancer cells. Halofuginone reduced the number only of stroma myofibroblasts expressing both contractile and collagen biosynthesis markers. Both stroma myofibroblasts and tumor cells generated ECM that contributes to tumor growth. Combination of treatments that inhibit stroma cell infiltration, cause apoptosis of myofibroblasts and inhibit Smad3 phosphorylation, with chemotherapy that increases tumor-cell apoptosis without affecting Smad3 phosphorylation was more efficacious than either treatment alone. More tumors developed in fibrotic than in normal pancreas, and prevention of tissue fibrosis greatly reduced tumor development.

**Conclusions:**

The utmost importance of tissue fibrosis and of stroma cells for tumor development presents potential new therapy targets, suggesting combination therapy against stroma and neoplastic cells as a treatment of choice.

## Introduction

Most solid tumors consist of a mixture of neoplastic and non-neoplastic cells, together with extracellular matrix (ECM) components. The microenvironment of a developing tumor comprises of proliferating tumor cells, stroma cells, blood vessels and infiltrating inflammatory cells. It is a unique environment, which emerges during tumor progression as a result of tumor/host interactions; it is created by, and at all times is shaped and dominated by the tumor, which orchestrates molecular and cellular events taking place in surrounding tissues. [1] This cellular microenvironment, which is distinct from the normal tissue environment, directly modulates tissue architecture, cell morphology and cell fate. [2], [3] The interactions among the ECM, stromal and tumor cells and the various cytokines embedded in the ECM contribute to the neoplastic phenotype. [4] The predominant stroma cells infiltrating tumors and responsible for ECM synthesis are myofibroblasts (cancer- or tumor-associated fibroblasts) that can switch from tumor-suppressing to tumor-promoting functions during carcinogenesis. [5]–[7] The importance of myofibroblasts in tumor progression was demonstrated by co-inoculation of tumor cells with myofibroblasts in breast [8], [9] and pancreas xenografts, [10] which resulted in increases in tumorigenicity and tumor size. Moreover, tumor stromal myofibroblasts were more effective in promoting carcinogenesis than equivalent fibroblasts extracted from noncancerous tissue of the same individual or from healthy donors. [11], [12] Collagen type I, the major ECM component produced by myofibroblasts, not only functions as a scaffold for the tissue but also regulates the expression of genes associated with cellular signaling and metabolism, and gene transcription and translation. Thus, it affects fundamental cellular processes that are essential for tumor progression, such as cell survival, apoptosis and cell invasion. [13]–[15] ECM in general, and collagen type I in particular, can promote epithelial–mesenchymal transformation (EMT) [16], [17], which is an additional source of myofibroblasts. [6], [18] Furthermore, in various malignancies, tumor-dependent transformation of fibroblasts to myofibroblasts enhances neoplastic progression, and the presence of desmoplastic stroma enriched in myofibroblasts was associated with unfavorable prognoses. [14], [19]–[21] The fibroblast-to-myofibroblast transition is driven especially by transforming growth factor-beta (TGFβ), secreted either by the stroma cells [22], or by the tumor itself via the cancer exosomes. [23] In addition to the increase in ECM synthesis, the fibroblasts that acquire an activated phenotype are characterized by expression of contractile genes such as α smooth-muscle actin (αSMA) and transgelin (SMA22α), and exhibit a highly proliferative and migratory phenotype. [24].

**Figure 1:**
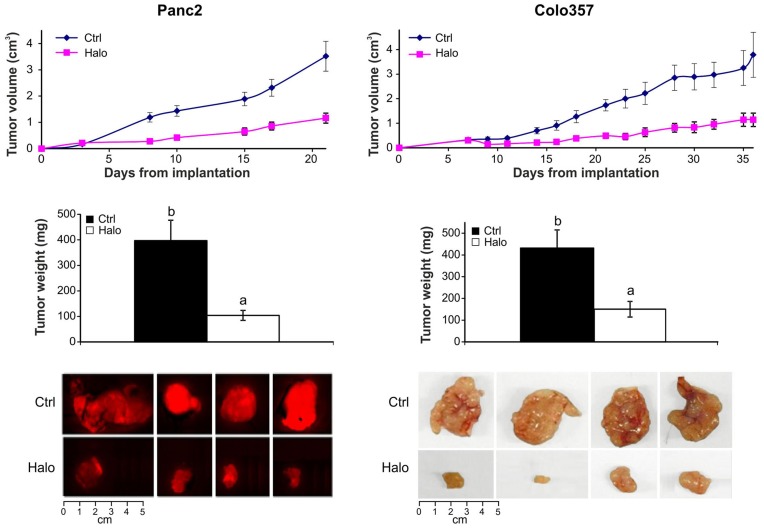
Inhibition of tumor development by halofuginone. Mouse (mCherry-labeled Panc2) and human (colo357) pancreatic tumor cells were transplanted subcutaneously into C57/GFP and nude mice, respectively. Halofuginone (15 µg/mouse) was injected ip three times weekly, starting 3 days post-transplantation. During tumor progression tumor size was monitored by caliper. After 21 days (Panc2) and 36 days (colo 357) the tumors were removed, weighted and photographed. Results are means ± SE and columns with different letters within each experiment differ significantly (*P*<0.05).

Stellate cells of the pancreas (PSC) and of the liver (HSC) constitute the major source of the ECM in the tumoral stroma. [25]–[27] These cells are usually quiescent, with a low proliferation rate; however, upon activation they differentiate into myofibroblast-like cells, which proliferate and migrate to tumor sites, where they synthesize ECM components and promote tumor progression. [28], [29] The SCs can be activated by hepatic [30] and pancreatic [28] tumors, and the cross-talk between tumor cells and the surrounding stroma is a key modulator of hepatocarcinogenesis. [31] The PSCs have been identified as the principal source of the excessive ECM production in chronic pancreatitis and pancreatic adenocarcinoma. [32] The crucial roles of the stroma myofibroblasts in tumor development mark them as a target for cancer therapy. [21].

In the present study we focused on the heterogeneity of the pancreatic stroma cells and the origin of the collagen within the tumors. We demonstrated the importance of cerulean–induced pancreas fibrosis for tumor development. By using halofuginone, an inhibitor of Smad3 phosphorylation downstream of the TGFβ signaling pathway, [33], [34] and known to inhibit HSC and PSC activation[35]–[37] and of the fibroblast-to-myofibroblast transition in the tumor microenvironment, [38], [39] we were able to identify reduction of myofibroblasts, especially in combination with chemotherapy, as a target treatment for reducing tumor growth.

**Figure 2:**
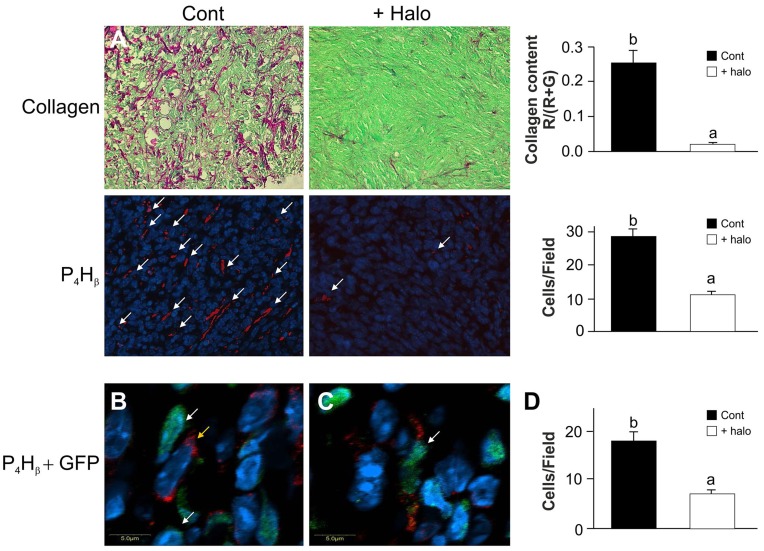
Collagen and P4Hβ-expressing cells in the tumor. A– Biopsies from panc2 subcutaneous tumors were stained with Sirius red for collagen (red) and immune-stained with P4Hβantibodies (red, indicated by arrows). Halofuginone treatment three times weekly at 15 µg/mouse resulted in major reductions in collagen content and in the total number of P4Hβ-expressing cells. B– Biopsies were double-immunostained with GFP-antibodies (green) and P4Hβ antibodies (red), and nuclei were stained with DAPI (blue). A stroma cell not expressing P4Hβ (white arrow) is adjacent to a tumor cell expressing P4Hβ (yellow arrow). C– Stroma cell expressing P4Hβ (white arrow). D– Halofuginone treatment reduced the total number of stroma cells expressing P4Hβ. Results are means ± SE and columns with different letters within each experiment differ significantly (*P*<0.05).

**Figure 3:**
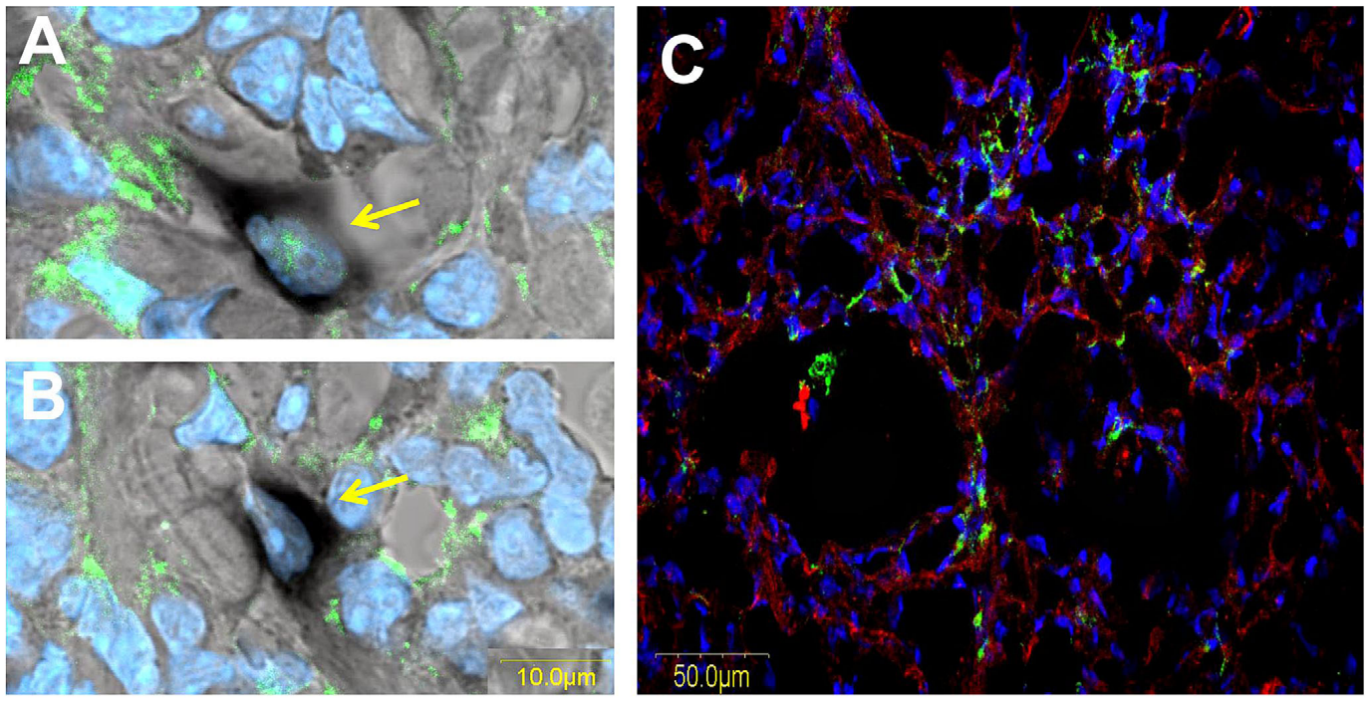
Origin of the tumor collagen. A & B– Biopsies of subcutaneous panc2 tumors 21 days post-transplantation were hybridized with collagen type I probe (black) and were immunostained with GFP antibodies (green). Nuclei were stained with DAPI (blue). Arrows point to: A– cell expressing GFP and mRNA for collagen α1(I); B– cell expressing mRNA for collagen α1 but not GFP; C– Subcutaneous tumors were established by implanting 10^6^ human MiaPaca2 into male C57/GFP mice. Biopsies were immunostaining with human (green) and mouse (red) collagen antibodies. Collagen from both human and mouse origin is observed within the tumor.

## Results

### Inhibition of Tumor Growth and Development by Halofuginone

Subcutaneous pancreatic tumors were established in immuno-non-compromised C57/GFP mice implanted with a mouse cancer cell line (Panc2 labeled with mCherry) and in immuno-compromised mice implanted with a human cancer cell line (colo357). Halofuginone treatment resulted in significant (*p*<0.005) reductions in tumor growth already after 8 and 14 days in the mouse and human tumors, respectively. At the end of the experiments, the tumor weight and size were significantly (*p*<0.005) lower in the halofuginone-treated mice than in the controls (Fig. 1).

**Figure 4:**
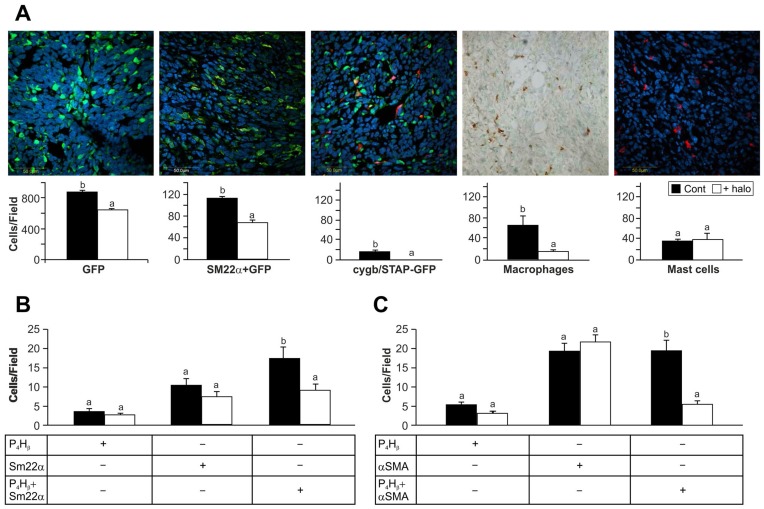
Effect of halofuginone on host cells migrating into the tumor. Biopsies of subcutaneous panc2 tumors 21 days post-transplantation, with and without halofuginone treatment three times weekly at 15 µg/mouse, were: A– stained with GFP antibodies or double-stained with GFP plus SM22α, GFP plus Cygb/STAP, or macrophages plus mast cell antibodies; Halofuginone inhibited the number of total stroma cell within the tumor and reduced the number of macrophages and myofibroblasts expressing SM22α, Cygb/STAP but not the number of mast cells. B– immunostained with P4Hβ or SM22α antibodies or double-stained with both antibodies; C– immunostained with P4Hβ or αSMA antibodies or double-stained with both antibodies. Halofuginone inhibited the number of stroma cells expressing both the collagen cross-linking enzyme and smooth muscle genes. At least 20 photographs of tissue sections from different mice were taken for each analysis. Results are means ± SE and columns with different letters within each experiment differ significantly (*P*<0.05).

**Figure 5:**
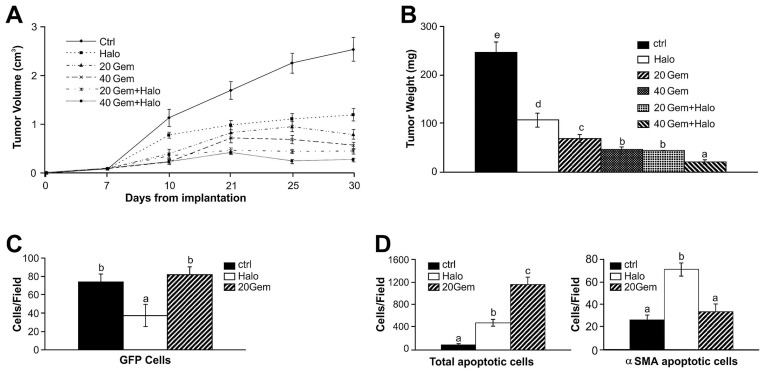
Combination therapy. Mouse pancreatic tumor cells (panc2) were implanted subcutaneously and treated with two doses of gemcitabine, alone or in combination with halofuginone. A– Tumor volume during 30 days. B– Tumor weight at the end of the experiment. C– Effects of halofuginone and gemcitabine on the number of host cells in the tumor. D– Effects of halofuginone and gemcitabine on apoptosis of all cells within the tumor, or of the cells that expressed αSMA. Results are means ± SE and columns with different letters within each experiment differ significantly (*P*<0.05).

**Figure 6:**
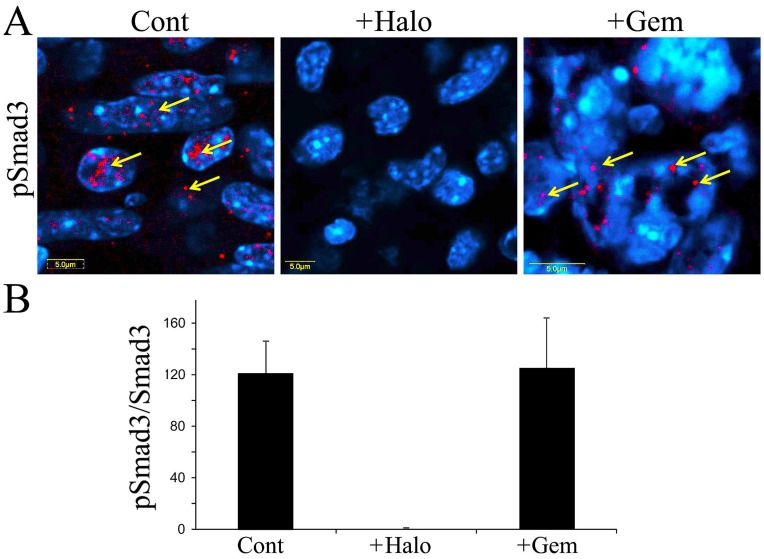
Mouse pancreatic tumor cells (panc2) were implanted subcutaneously and treated with either halofuginone three times weekly at 15 µg/mouse or gemcitabine twice weekly at 20 mg/kg, starting 7 days after tumor cell implantation. At the end of the experiment, i.e., 30 days after cell implantation: A– tumors were taken for immunohistochemistry with pSmad3 antibodies; B– at least 20 photographs from five different mice from each group were taken for each analysis. The results are expressed as arbitrary pSmad3/Smad units ± SE. Results are means ± SE and columns with different letters within each experiment differ significantly (*P*<0.05).

### Origin of the Tumor ECM

The ECM is pivotal for cancer cell growth, tumor invasion, and metastatic progression. [18] Pancreatic tumors derived from panc2 cells contained high levels of collagen (stained red) and were populated with cells expressing prolyl 4 hydroxylase β(P4Hβ), the major collagen cross-linking enzyme (Fig. 2A). Halofuginone treatment resulted in major reductions in collagen content and in the number of P4Hβ-expressing cells. These results are consistent with previous findings of halofuginone-dependent inhibition of collagen synthesis in various xenografts.[10], [38], [40]–[42] Most (64%) of the P4Hβ-expressing cells within the tumor derived from invading host cells (GFP-positive) and the remaining 36% (GFP-negative) derived from the tumor, probably via EMT. Halofuginone treatment reduced the numbers of P4Hβ-expressing cells in both the stroma and the tumor (Fig. 2B–D).

**Figure 7:**
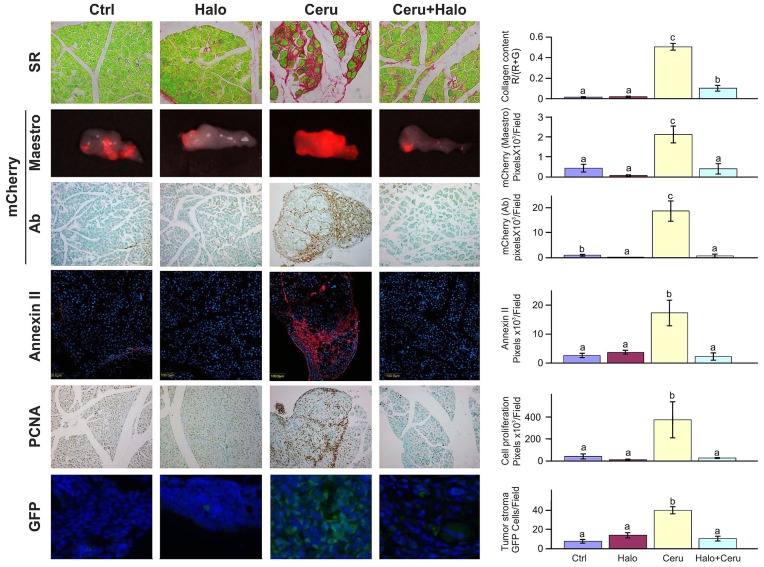
Pancreatic fibrosis and tumor development. C57/GFP mice were treated with halofuginone or cerulein, alone or in combination, and untreated mice served as controls. After 8 weeks, pancreatic fibrosis was validated by Sirius red (SR) staining and quantified by image analysis. At that time, the cerulein and halofuginone treatments were terminated, and mCherry-labeled panc2 cells were injected into the spleen; after a further 2 weeks, mouse pancreas was photograph with the Maestro imaging system. Pancreas sections were stained with mCherry, annexin II, PCNA and anti-GFP antibodies, and positive signals were quantified. In the fibrotic tissue more stroma (GFP-positive) and tumors cells (annexin II–positive) were observed. Halofuginone treatment that lessens tissue fibrosis reduced both the tumor and stroma cell number. Results are means ± SE and columns with different letters within each experiment differ significantly (*P*<0.05).

To elucidate the origin of tumor collagen, biopsies from subcutaneous panc2 tumors were hybridized with a collagen type I probe, following immunostaining with anti-GFP antibodies (Fig. 3). The cells that were positive for collagen α1(I) mRNA comprised both GFP-expressing cells that originated from the host (Fig. 3A) and GFP-non-expressing cells that originated from the tumor (Fig. 3B). To further confirm these results, tumor biopsies derived from human pancreatic cancer cells (MiaPaca2) implanted in nu/nu mice were double-stained with human- and mouse-specific collagen type I antibodies (Fig. 3C). The species specificity of the antibodies was validated by using human keloids and mouse fibrotic pancreas, which are rich in human and mouse collagen type I, respectively (data not shown). Both human and mouse type I collagens were found in the tumors (Fig. 3C), the mouse being by far the major one –76% mouse collagen compare to of 24% human one.

### Host Cells Invading the Tumor

The C57/GFP mice are ideal for evaluating the numbers and types of host cells invading the tumor. Subcutaneous tumors that developed after implantation of panc2 cells contained numerous host cells, as indicated by presence of GFP antibodies (Fig. 4A). Halofuginone treatment resulted in a significant (*p*<0.025) decrease in the host-cell population within the tumor. To determine whether the halofuginone targeted a specific cell population we compared the numbers of myofibroblasts, macrophages and mast cells in the tumor, with and without halofuginone treatment. Acquisition of an activated phenotype of fibroblasts is associated with expression of markers such as smooth muscle genes (αSMA and SM22α)), enzymes involved in collagen biosynthesis (P4Hβ), and oxidative-stress genes like stellate cell activation-associated protein (Cygb/STAP). Halofuginone treatment reduced the numbers of stroma myofibroblasts expressing SM22α or Cygb/STAP, and the number of macrophages, but not that of mast cells. The myofibroblasts within the tumor were not a homogeneous population: some expressed only P4Hβ, αSMA, or SM22α; others expressed more than a single marker (Fig. 4B). Halofuginone decreased the numbers only of cells that expressed a smooth muscle marker (αSMA or SM22α) together with P4Hβ, the collagen-cross-linking enzyme. In the tumor all the cells expressing SM22α derived from the host, whereas only 62% of the cells expressing αSMA were GFP-positive, with the remaining 38% derived from the tumor.

### Combination Therapy

Thanks to its unique mode of action, halofuginone is ideal for combination therapy. [38] The C57/GFP mice were subcutaneously implanted with panc2 mouse pancreatic cancer cells and then were treated with halofuginone and with high or low doses of gemcitabine, alone or in combination. Either halofuginone or gemcitabine, administered alone, inhibited development of the tumors, but treatment with the combination of the two was more efficacious than either of them alone. Halofuginone with the low dose of gemcitabine achieved a significant reduction in tumor volume and weight, comparable with the effect of the high dose of the chemotherapy; tumors barely developed in mice treated with halofuginone combined with the high dose of gemcitabine (Fig. 5A). At the end of the experiment tumors were excised and weighed (Fig. 5B): treatment with halofuginone or either dose of gemcitabine reduced tumor weight, the high dose being more efficacious than the low dose, but the combination therapy was superior to use of any of the drugs alone. We evaluated the ability of halofuginone and gemcitabine to inhibit tumor invasion by stroma cells and to cause apoptosis, and found that only halofuginone, but not gemcitabine, reduced the number of GFP-stroma cells infiltrating the tumors (Fig. 5C). Both halofuginone and gemcitabine increased apoptosis (Fig. 5D), but halofuginone specifically increased apoptosis of αSMA-expressing cells, whereas gemcitabine did not affect it (Fig. 5E). Only halofuginone, but not gemcitabine, inhibited phosphorylation of Smad3 downstream of the TGFβ signaling pathway in the pancreatic tumors (Fig. 6) without affecting the levels of total Smad3 (data not shown).

### Tissue Fibrosis and Tumor Development

Activated SC constitute the main production source of the ECM that is essential for tumor establishment and growth. [43], [44] In order to evaluate the significance of tissue fibrosis for pancreatic tumor growth and development, we induced pancreatic fibrosis in the C57/GFP mice by application of cerulein, with and without halofuginone. Fibrosis was determined in the pancreas after 8 weeks by staining the tissue biopsies with Sirius red. Cerulein induced fibrosis 37-fold, and halofuginone significantly (*p*<0.05) reduced pancreas collagen levels, in agreement with our previous findings. [35], [37] After confirmation that fibrosis had occurred, the halofuginone and cerulein treatments were terminated, and tumor cells (mCherry-labeled panc2) were injected into the spleens of the mice with and without pancreatic fibrosis. After a further 2 weeks, the animals were sacrificed and the pancreatic tissue was subjected to histopathology. In the pancreas of the control mice almost no collagen was detected, the tumors were small, as evaluated by the ex*-vivo* imaging fluorescence-based detection system and by immunostaining with mCherry antibodies, and hardly any proliferating cells (PCNA-positive), GFP-stroma cells or annexin II-expressing cells were detected in the tumors (Fig. 7). Annexin II is part of a tumor-host signal pathway involved in degradation of ECM therefore it plays an important role in the tumor microenvironment. Up-regulation of annexin II levels was observed in HCC and pancreatic tumors, whereas minimal expression was observed in normal tissues. [45], [46] In the control mice halofuginone administration did not elicit any changes in pancreas collagen levels, in the number of host cells, or in annexin-expressing cells; fewer mCherry-labeled cells were detected by immunostaining but not by the ex-*vivo* optical fluorescence-based imaging detection system. Cerulein treatment elicited a major increase in pancreatic fibrosis, and the tumors that proliferate along the ECM fibers occupied almost the entire pancreas; the numbers of annexin II-positive cells, proliferating cells and GFP-positive stroma cells increased by six-, nine- and fivefold, respectively. Prevention of pancreatic fibrosis by halofuginone resulted in major decreases in tumor volume; the tumors contained hardly any proliferating and annexin-positive cells, and were devoid of GFP-host cells. It is important to note that the tumors that developed in the fibrotic pancreas exhibited a higher level of collagen than those that developed in the normal pancreas, and that this high level remained throughout the experiment without any resolution. The tumor collagen was reduced almost to control levels by halofuginone treatment (data not shown).

## Discussion

Various tumors are characterized by abundant, dense, reactive stroma, and the interaction of malignant cells with non-parenchymal stromal cells is crucial for cancer progression. [47] In the present study a variety of host cells that populated the tumor included cells of the inflammatory systems, such as macrophages and mast cells, and myofibroblasts (Fig. 4). The myofibroblasts that play a central role in the tumor–stroma crosstalk, comprise heterogeneous and multifunctional cell populations that exhibit various phenotypes, [5], [19] and express divers markers. [48], [49] By using ubiquitin GFP-positive transgenic mice we were able to identify host myofibroblast subpopulations, some of which expressed only contractile markers (αSMA; SM22α), markers involved in collagen biosynthesis (P4Hβ), or oxidative-stress gene (Cygb/STAP), and some of which expressed more than one marker. This suggests that there are subpopulations of myofibroblasts, whose specific phenotypes might be determined by the developmental stage of the tumor, by differing origins and lineages, or by site-specific differentiation. For example, a subset of human pancreatic cancer myofibroblasts was identified as being particularly relevant to tumor growth, and was found to be actively recruited and induced, to provide support to the tumor. [50] Halofuginone, which inhibited tumor growth and development (Fig. 1) and reduced P4Hβ and collagen levels (Fig. 2), reduced the number of myofibroblasts, but only of those that expressed both the contractile and the collagen-biosynthesis markers (Fig. 4). The specificity of halofuginone can be attributed, in part, to the heterogeneous TGFβ signaling of stromal cells and to the loss of TGFβ responsiveness in the subpopulation of stromal cells that induces tumorigenicity. [51] EMT-derived mesenchymal cells are another subset of cells, some of which express αSMA, but none express transgelin at this stage of tumor development.

In the pancreatic tumors, cells derived either from the host (GFP-positive) or from the tumor (GFP-negative) – probably via EMT – expressed the collagen α1(I) gene (Fig. 3A, B) and were responsible for the human and mouse collagen type I within the tumor (Fig. 3C). Both the TGFβ secreted by the host inflammatory cells that induce the fibroblast-to-myofibroblast transition [21] and the collagen type I synthesized by the myofibroblasts [52] can promote EMT and thereby elicit a further increase in collagen levels. Moreover, activated myofibroblasts can induce tumor progression in a TGFβ-dependent fashion, [53] and this vicious circle, which involves the TGFβ/Smad3 pathway, is crucial for tumor development. Halofuginone that inhibited Smad3 phosphorylation (Fig. 6) reduced the number of host cells invading the tumor (Fig. 4A), collagen biosynthesis (Fig. 3), and tumor growth (Fig. 1).

Marked stromal fibroblast proliferation and deposition of ECM components, driven by the TGFβ/Smad signaling pathway, form one of the hallmarks of pancreatic ductal adenocarcinoma (PDAC), which imply invasive progression of the tumor and an unfavorable prognosis. [25], [26] The importance of tissue fibrosis for tumor growth and development was evaluated in chemically induced pancreatic fibrosis (Fig. 7). Increased fibrosis resulted in increases in numbers of tumors, high levels of annexin II and, also in presence of mCherry in the pancreas. The tumors established in the fibrotic tissues contained large numbers of stroma cells, but after inhibition of tissue fibrosis by halofuginone far fewer tumors were established, and those that remained were smaller and almost devoid of stroma cells.

Not only is the stroma important for tumor progression; an extensive stromal component of the tumors has been hypothesized to influence the response of pancreatic tumors to chemotherapy [54], [55] and the PSC-mediated inhibition of pancreatic cancer cell responses to gemcitabine. [12] Thus, a combination of anticancer treatments that exhibit differing modalities and simultaneously target both the malignant cells and their stroma cell-populated microenvironment, with minimal somatic and genetic alterations, could form a therapy of choice. Thanks to its unique mode of action, which involves inhibition of Smad3 phosphorylation (Fig. 6), halofuginone is an ideal candidate for combination therapy. Only the halofuginone treatment, and not the chemotherapy, inhibited the tumor invasion by stroma cells (Fig. 5C) and increased apoptosis of the αSMA-positive cells (Fig. 5D). It should be noted that αSMA is a marker of aggressiveness in some tumors. [56] Moreover, halofuginone inhibited the tumor-recruited macrophages (tumor-associated macrophages – TAMs), which are known to be an important component of the tumor microenvironment and to promote tumor progression, and which correlate with unfavorable prognoses. [57] On the other hand, mast cells, which modulate the tumor microenvironment, were not affected by halofuginone (Fig. 4), as was observed in the tight-skin (Tsk) mouse model of scleroderma [58]; this indicates the specificity of halofuginone targets.

Administration of halofuginone together with chemotherapy resulted in inhibition of tumor development that was superior to the effect of either drug alone (Fig. 5A, B). Moreover, low dose of halofuginone together with low dose of chemotherapy were as efficacious as the high chemotherapy dose which suggests a possible reduction in the overall treatment burden and in the chemotherapy-related side effects. Similar results were observed in prostate cancer and Wilms’ tumor: halofuginone, but not the respective chemotherapies, inhibited synthesis of collagen type I, αSMA, SM22α, and Cygb/STAP, all of which are characteristic of activated myofibroblasts. [38].

Mouse models of cancer have consistently been used to qualify new anti-cancer drugs for development of human clinical trials. The most used models are xenografts of human tumors grown subcutaneously in immunodeficient mice (nu/nu) or severe combined immune deficient (SCID) mice. The major limitations of these models are a lesser involvement of the immune system, different microenvironment and dissimilar vasculature. On the other hand they are easy to execute and it is simple to follow tumor growth. Alternatively, the orthotopic transplantation model that promotes metastatic spread and growth of the tumor cells in a physiological relevant site are thought to represent a more clinically relevant tumor model. One of the most obvious advantages of orthotopic systems is that attempts to target processes involved in local invasion and angiogenesis can be carried out in a more clinically relevant site. It is important to note that the significance of other components of the ECM for tumor development was demonstrated using genetically engineered mouse models bearing autochthonous tumors that recapitulate the human pathology [59], [60]. In addition, disrupting the stroma of pancreatic tumors altered the vascular network and thereby facilitated the delivery of chemotherapeutic agents. [61] The tumors of PDAC patients consist of well established fibrotic stroma [62]. Halofuginone, in addition to preventing fibrosis can also cause resolution of existing fibrosis as demonstrated in various animal models representing various fibrotic conditions [35], [58], [63] and in humans [64]. We cannot rule out the possibility that in addition to inhibition of Smad3 phosphorylation down-stream of the TGFβ signaling which is probably the canonical mode of action of halofuginone, it may affect also other pathways such as the amino acid starvation response [65] and inhibition of prolyl-tRNA synthetase activity [66] as well.

In summary, pancreatic tumors consist of cancer cells and heterogeneous populations of stroma cells – mainly myofibroblasts, and macrophages. Both the stroma myofibroblasts and the cancer cells that undergo EMT are responsible for the intra-tumoral ECM. Inhibition of the TGFβ/Smad3 pathway inhibits stroma cell invasion and tumor growth. Pancreatic fibrosis gives rise to a massive infiltration of the tumor by stroma cells, which is of great importance for successful of tumor development. Thus, a therapy that targets stroma cell invasion and apoptosis, combined with chemotherapy that targets the neoplastic cells results in almost complete arrest of the tumor.

## Materials and Methods

### Materials

Halofuginone was obtained from Halo Therapeutics LLC (Newton, MA, USA), and gemcitabine hydrochloride (GEMZAR) from Eli Lilly and Company (Indianapolis, IN, USA). Antibodies to stellate cell activation-associated protein (cygb/STAP) were a gift from Dr. Norifumi Kawada (Osaka University, Japan). Monoclonal human and polyclonal mouse-specific collagen type I, monoclonal F40/80 macrophages, polyclonal transgelin (SM22α) and polyclonal annexin II antibodies were obtained from Abcam (Cambridge, UK); polyclonal GFP antibody from MBL International (Woburn, MA, USA); monoclonal GFP, polyclonal tryptase, polyclonal Smad3 and pSmad3 antibodies from Santa Cruz Biotechnology (Santa Cruz, CA, USA); monoclonal biotin anti-PCNA from BioLegend (San Diego, CA, USA); monoclonal α smooth muscle actin (αSMA) antibodies from Dako (Santa Barbara, CA, USA); polyclonal mCherry antibodies from BioVision (Aachen, Germany); and prolyl 4 hydroxylase β (P_4_Hβ) polyclonal antibodies from Proteintech Group (Chicago, IL, USA). MiaPaca2, Colo357 and Panc2 cell lines were purchased from American Type Culture Collection (ATCC, Manassas, VA, USA).

### Animals and Experimental Design

All animal experiments were carried out in accordance with the guidelines of the Volcani Center Institutional Committee for Care and Use of Laboratory Animals (Inhibition of stroma cell invasion and matrix proteins as a novel modality for cancer therapy -IL358/11). The C57/GFP (ubiquitin-EGFP mice, Jackson Laboratory C57BL/6-Tg[UBC-GFP]30Scha/J) that express enhanced GFP under the direction of the human ubiqutin C promoter in all tissues and the expression is uniform within a cell type lineage and remains constant throughout development and nu/nu mice (Harlen Laboratories, Israel) were housed in cages under conditions of constant photoperiod (12 L:12 D) with free access to food and water. Subcutaneous tumors were established by implanting 10^6^ human (MiaPaca2 or Colo 357; *n = *16 of each xenograft) and mouse (mCherry-labeled Panc2; *n = *14) pancreatic tumor cells into male nu/nu and C57/GFP mice, respectively. Halofuginone at 15 µg/mouse was injected intraperitoneally (ip) in saline, three times/week starting 3 days after cell implantation, and control animals were injected with saline. Combination therapy was evaluated by implanting 10^6^ mCherry-labeled Panc2 cells into C57/GFP mice (*n = *9 mice/group), ip injection of gemcitabine at 20 or 40 mg/kg twice weekly, and halofuginone at 15 µg/mouse three times weekly, starting 7 days after tumor cell implantation. Tumor size was determined with a caliper, as length × width × depth × 0.5236, and is presented as mean ± SE.

To evaluate the importance of tissue fibrosis for tumor development pancreatic fibrosis (*n* = 10 mice/group) was induced in C57/GFP mice by repeated 4-hourly ip injections of cerulein (50 µg/kg) in saline for 4 weeks followed by 6-hourly injections for an additional 4 weeks. [10], [37] After 8 weeks, when pancreatic fibrosis, was established, mice were anesthetized (12 mg/kg xylazine and 100 mg/kg ketamine) after which a surgical incision of the skin and peritoneum lateral to the mid section was made under sterile conditions and a dose of 10^6^ Panc2 cells was injected directly into the spleen. After an additional 2 weeks the animals were sacrificed and the pancreatic tissues were taken for histopathology. Halofuginone was injected ip at 15 µg/mouse three times weekly, starting with the cerulein and the treatment was terminated before tumor cell implantation. The Maestro noninvasive fluorescence imaging system (CRI Inc, Hopkinton, MA, USA) was used to evaluate mCherry-labelled subcutaneous and pancreatic tumors.

### Preparation of Sections, Immunohistochemistry and *in situ* Hybridization

Biopsies were collected and fixed overnight: in 4% paraformaldehyde in PBS at 4°C for Sirius red staining; in Bouin solution for combination therapy; and without fixation for the human and mouse collagen. For immunohistochemistry frozen and paraffin-embedded tissue sections were used: serial 5-µm sections were prepared and embedded in Paraplast, and the paraffin sections were stained with Sirius red for collagen and counter-stained with fast green. Collagen levels were quantified by image analysis with Photoshop software, and the number of labeled cells in each field was quantified with NIS-Elements AR 3.2 software (Melville, NY, USA). At least 20 photographs of tissue sections from different mice were taken for each analysis; the results were calculated as red (R) area divided by the total (red + green) (R+G) area, and presented as arbitrary units of the mean ± SE. Pancreatic tumors were identified by evaluation of cell proliferation (PCNA) and annexin II level. Immunohistochemistry was performed with the following antibodies: human and mouse collagen (1∶50); Cygb/STAP (1∶250); P_4_Hβ (1∶200); αSMA (1∶200); SM22α (1∶200); annexin II (1∶200); mouse and rabbit anti-GFP (1∶400); PCNA (1∶500); tryptase (1∶100); Smad3 and pSmad3 (1∶50), F4/80 (1∶150) Smad3 and pSmad3 (1∶1000) antibodies and mCherry (1∶150). Nuclei were stained with 4′,6-diamidino-2-phenylindole dihydrochloride (DAPI). *In situ* hybridization for collagen α1(I) was performed as previously described [37] and was followed by immunohistochemistry with monoclonal GFP antibodies. The number of apoptotic cells was evaluated with the MEBSTAIN Apoptosis Kit (MBL International, Woburn, MA, USA).

### Confocal Microscopy

Image acquisition was performed with an Olympus IX 81 inverted confocal laser-scanning microscope (Fluoview 500; Olympus, Tokyo, Japan). DAPI, “Green” and “Red” were excited at 405, 488 and 543 nm, and the emission was collected through BA 430–460, 505–525, and 560 filters, respectively. Primary antibodies were visualized as follows: Goat anti-mouse IgG antibody with Alexa Fluor dye (Molecular Probes, Carlsbad, CA, USA); Donkey anti-mouse IgG antibody with Cy3; Donkey anti-rabbit IgG with Cy2; Donkey anti-goat with Cy2; and Donkey anti-rabbit with Cy3 (Jackson Immunoresearch, West Grove, PA, USA).

### Statistical Analysis

Data were subjected to one-way analysis of variance (ANOVA) and to the all-pairs Tukey-Kramer HSD test by means of the JMP software.
